# Glucose-Dependent Insulinotropic Polypeptide Mitigates 6-OHDA-Induced Behavioral Impairments in Parkinsonian Rats

**DOI:** 10.3390/ijms19041153

**Published:** 2018-04-11

**Authors:** Yu-Wen Yu, Shih-Chang Hsueh, Jing-Huei Lai, Yen-Hua Chen, Shuo-Jhen Kang, Kai-Yun Chen, Tsung-Hsun Hsieh, Barry J. Hoffer, Yazhou Li, Nigel H. Greig, Yung-Hsiao Chiang

**Affiliations:** 1 The Ph.D. Program for Neural Regenerative Medicine, College of Medical Science and Technology, Taipei Medical University, Taipei 11031, Taiwan; yvonneyu524@hotmail.com (Y.-W.Y.); d620103001@tmu.edu.tw (S.-C.H.); chenkathryn@hotmail.com (K.-Y.C.); hsiehth@mail.cgu.edu.tw (T.-H.H.); ychiang@tmu.edu.tw (Y.-H.C.); 2 Center for Neurotrauma and Neuroregeneration, Taipei Medical University, Taipei 11031, Taiwan; m105095006@tmu.edu.tw (J.-H.L.); swallows3366@gmail.com (Y.-H.C.); terbiun@gmail.com (S.-J.K.); 3 Department of Surgery, School of Medicine, College of Medicine, Taipei Medical University, Taipei 11031, Taiwan; 4 Department of Physical Therapy and Graduate Institute of Rehabilitation Science, College of Medicine, Chang Gung University, Taoyuan 33302, Taiwan; 5 Department of Neurosurgery, Case Western Reserve University School of Medicine, Cleveland, OH 44106, USA; 6 Drug Design & Development Section, Translational Gerontology Branch, Intramural Research Program, National Institute on Aging, National Institutes of Health, Baltimore, MD 20892, USA; yazhou.Li@nih.gov (Y.L.); greign@grc.nia.nih.gov (N.H.G.); 7 Department of Neurosurgery, Taipei Medical University Hospital, Taipei 11031, Taiwan

**Keywords:** glucose-dependent insulinotropic polypeptide, 6-hydroxydopamine, Parkinson’s disease, neuroprotection, incretin

## Abstract

In the present study, the effectiveness of glucose-dependent insulinotropic polypeptide (GIP) was evaluated by behavioral tests in 6-hydroxydopamine (6-OHDA) hemi-parkinsonian (PD) rats. Pharmacokinetic measurements of GIP were carried out at the same dose studied behaviorally, as well as at a lower dose used previously. GIP was delivered by subcutaneous administration (s.c.) using implanted ALZET micro-osmotic pumps. After two days of pre-treatment, male Sprague Dawley rats received a single unilateral injection of 6-OHDA into the medial forebrain bundle (MFB). The neuroprotective effects of GIP were evaluated by apomorphine-induced contralateral rotations, as well as by locomotor and anxiety-like behaviors in open-field tests. Concentrations of human active and total GIP were measured in plasma during a five-day treatment period by ELISA and were found to be within a clinically translatable range. GIP pretreatment reduced behavioral abnormalities induced by the unilateral nigrostriatal dopamine (DA) lesion produced by 6-OHDA, and thus may be a novel target for PD therapeutic development.

## 1. Introduction

Parkinson’s disease (PD) is the second most common neurodegenerative disease and is characterized by symptoms related to progressive dopamine neuron loss within the substantia nigra pars compacta [1]. In addition to dopaminergic neuron loss, other neurotransmitter systems have been indicated to be involved in the disease; thus, PD is now been thought to be multisystem and multisite disorder [2,3].

Previous research has demonstrated that treatment with glucagon-like peptide-1 (GLP-1), an endogenous incretin, as well as long-acting GLP-1 analogues, provide neurotrophic and neuroprotective actions in many acute and chronic neurodegenerative models through GLP-1 receptor (R) activation within the brain [4]. The activation of the GLP-1R on neurons induces potent neurotrophic and neuroprotective actions in cellular and animal models of neural injury and neurodegeneration [5,6,7,8,9,10], including models of PD [7,11,12]. Specifically, GLP-1 receptor (GLP-1R) agonists have demonstrated favorable activity in cellular and animal models of PD [5,13,14]. Indeed, there is an ongoing clinical trial with the nonhydrolyzable GLP-1 agonist Ex-4 in Parkinson’s disease [15,16].

**Figure 1:**
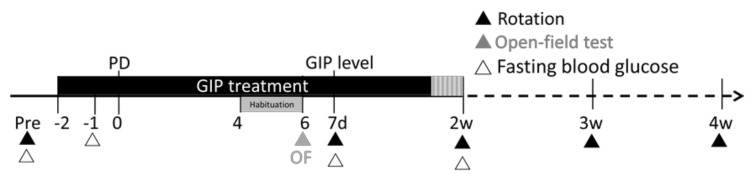
Schematic diagram of behavioral assessments and biochemical analyses of hemi-parkinsonian rats treated with a vehicle or GIP. GIP or saline was delivered s.c. via implanted micro-osmotic pumps for two weeks inserted two days prior to a unilateral 6-OHDA-induced dopamine lesion. Open-field tests were performed on day four after the lesion. The apomorphine-induced rotation was evaluated weekly during the four-week post-lesion period to identify the time-course of any effects of GIP treatment. Fasting blood glucose levels were observed prior to and during GIP treatment. GIP levels were monitored in a separate group of animals. PD: Parkinson’s disease. d: days; w: weeks.

Glucose-dependent insulinotropic polypeptide (GIP) is a second incretin that, like GLP-1, stimulates glucose-dependent insulin secretion, increases insulin biosynthesis, and improves β-cell proliferation and survival via its cognate receptor [17]. Combined with GLP-1, GIP has been proposed as a treatment strategy for type 2 diabetes mellitus [18,19]. Importantly, GIP, similar to GLP-1 and its agonists, promotes synaptic plasticity via its reversal of hippocampal long-term potentiation (LTP) impairments [20] and has neuroprotective and neurotrophic properties [21], suggesting a potential for halting or reversing neurodegenerative disorders. The GIP-R is also highly expressed across a number of CNS neurons [5,6], and GIP readily enters the brain [6,22]. GIP, however, has been much less studied in animal models of neurodegeneration. We have previously shown the neuroprotective activity of GIP in an animal model of traumatic brain injury [23]. We now report the efficacy of GIP in a rat model of PD induced by intracranial unilateral injection of the neurotoxin 6-OHDA, using behavioral tests. We have also carried out correlative measurements of GIP levels in plasma by ELISA at the same doses studied behaviorally (Figure 1).

## 2. Results

### 2.1. Body Weight and Fasting Blood Glucose Levels Are Not Affected by GIP Treatment

**Figure 2:**
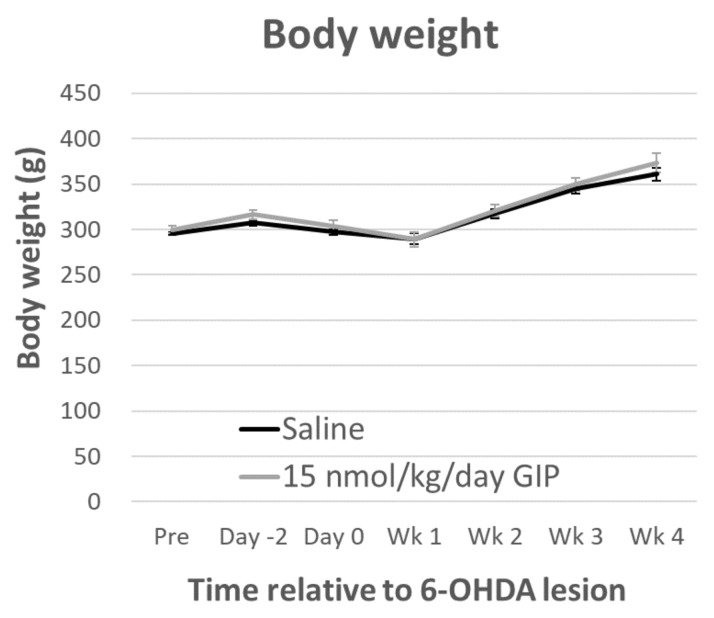
The average body weight of both groups were similar. 6-OHDA lesion caused body weight loss then recovery of a steady state with no treatment effects. All data are presented as mean ± SEM. (Saline: *n* = 12, 15 nmol/kg/day GIP: *n* = 11).

**Figure 3:**
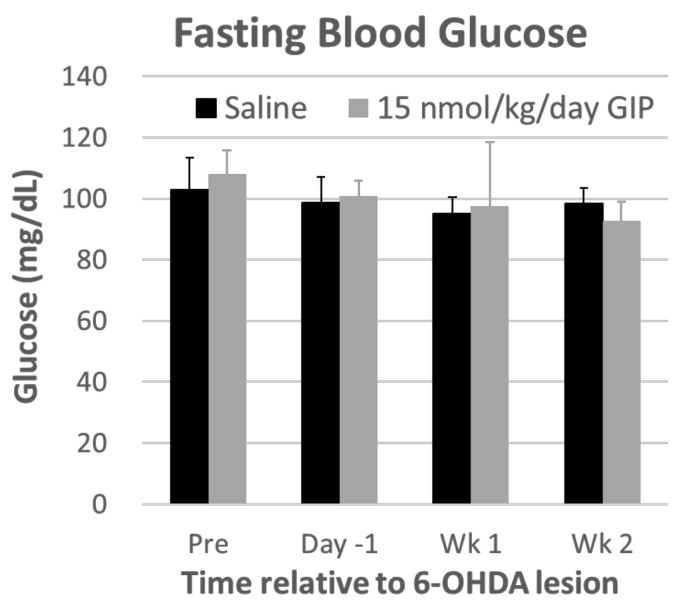
After an overnight (12 h) fast, blood glucose was measured by glucometer in tail vein blood and showed no change during GIP treatment. (*n* = 3/group).

Body weight: Repeated-measures ANOVA showed a significant effect of time (*F*(5, 100) = 89.650, *p* < 0.001) and a treatment × time interaction (*F*(1, 20) = 8661.285, *p* < 0.001), in rats one week after the 6-OHDA lesion. The body weights then returned to a steady-state and they did not differ between two treatments groups (*F*(1, 20) = 0.681, *p* = 0.419, Figure 2). After an overnight (12 h) fast, blood glucose was measured by glucometer in tail vein blood and showed no change during GIP treatment (Figure 3).

### 2.2. GIP Plasma Concentrations Are Significantly Elevated Following Steady-State Subcutaneous GIP Administration

**Figure 4:**
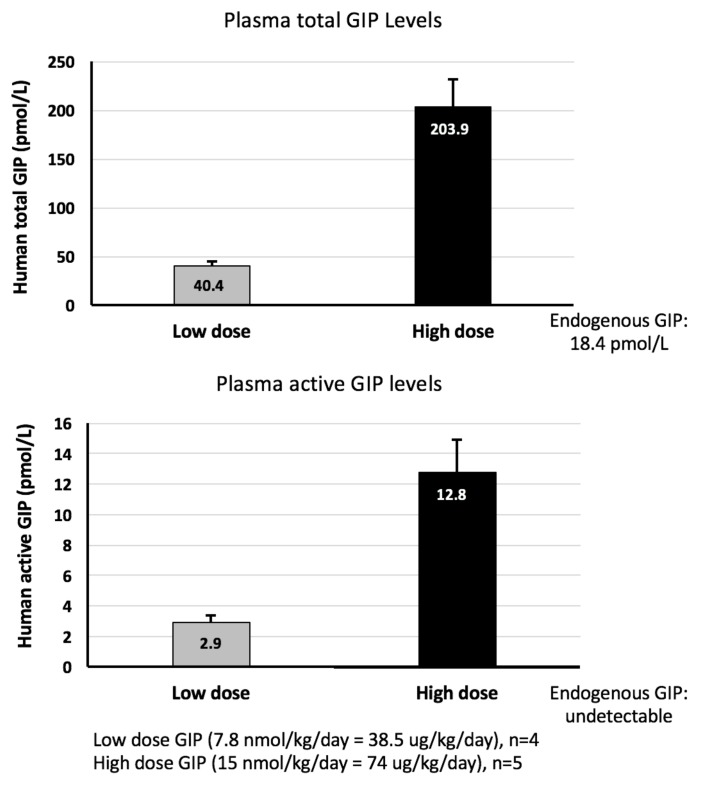
Plasma concentrations of total and active GIP were elevated above endogenous levels following steady-state administration of GIP (high dose: 15 nmol/kg/day; low dose: 7.8 nmol/kg/day). Upper panel: total GIP; plasma GIP concentrations from both high and low doses were significantly different from one another and were significantly elevated vs. endogenous levels (evaluated in saline administered animals) (*p* < 0.05). Lower panel: active GIP; plasma GIP levels following low and high doses were significantly different from one another (*p* < 0.05).

GIP plasma assays: In the present study, human GIP was assayed in plasma at two different doses in the Alzet pump, one of which used in the experiments described here and a second, lower dose used in our previous study on traumatic brain injury [23]. The total GIP levels included the bioactive GIP (1–42) and the non-insulinotropic N-terminally truncated metabolites GIP (3–42) both of which are actively degraded by the enzyme dipeptidyl peptidase IV (DPP-IV). The endogenous level of total GIP was 18.4 ± 2.1 pmol/L in the saline group, and the active GIP was undetectable in naïve rats with the saline administration. In a prior study [23], steady-state GIP administration of 7.8 nmol/kg/day, achieved by the ALZET mini pump, provided a plasma total GIP levels of 58.6 ± 11.8 pmol/L (2.2-fold elevation of control, *p* < 0.05), which is within the range of that measured currently (Figure 4, 40.4 pmol/L total GIP). Active GIP was measured for the first time in the current study (Figure 4). The 15 nmol/kg/day GIP’ group is just less than 2-fold higher than the 7.8 nmol/kg/day GIP and resulted in plasma total GIP levels that are 203.9 pmol/L (5-fold higher) and active GIP levels that are 4.4-fold higher.

### 2.3. GIP Reduces the Effects of Progressive Dopaminergic Pathway Dysfunction after 6-OHDA-Lesions

**Figure 5:**
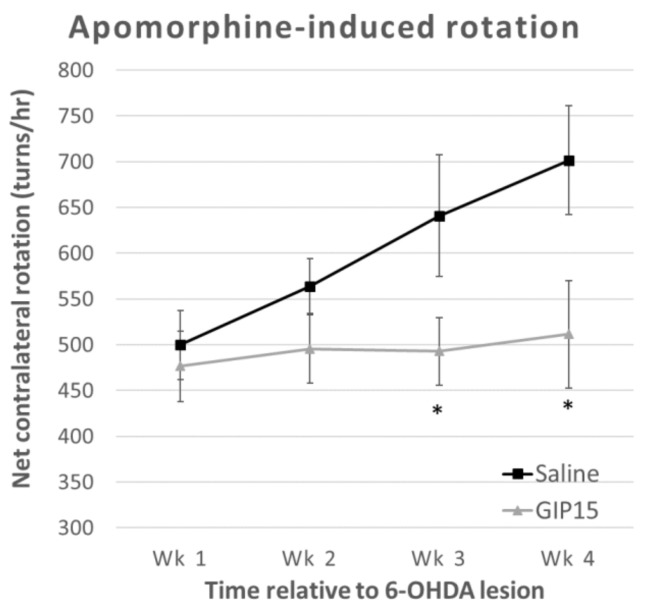
Apomorphine-induced rotation. The results obtained from apomorphine-induced rotational behavior tests after GIP vs. saline administration. GIP treatment shows statistically significant differences (* *p* < 0.05) compared to saline groups at week 3 and 4 post-6OHDA. (Saline: *n* = 9, GIP15: *n* = 10).

Apomorphine-induced rotation: To examine the effects of GIP on the modulation of dopaminergic transmission, we used apomorphine-induced contralateral rotations (Figure 5). A repeated measure two-way ANOVA found a difference both between groups (*F*(1, 17) = 6.132, *p* = 0.024) and over time (*F*(1.665, 28.302) = 3.674, *p* = 0.045), but no interaction effects (*F*(1.665, 28.302) = 2.699, *p* = 0.093). The main effect of time showed only differences in the saline group without GIP (saline: *F*(3, 24) = 4.138, *p* = 0.017; GIP: *F*(3, 27) = 0.132, *p* = 0.940). Furthermore, a two independent samples t-test revealed that there were significant differences between the saline and GIP groups on week 3 (*t* = 2.204, *p* = 0.042) and week 4 (*t* = 2.589, *p* = 0.019) after PD challenge.

### 2.4. The Effect of GIP on Changes in Open Field Behavior after a 6-OHDA-Lesion

**Figure 6:**
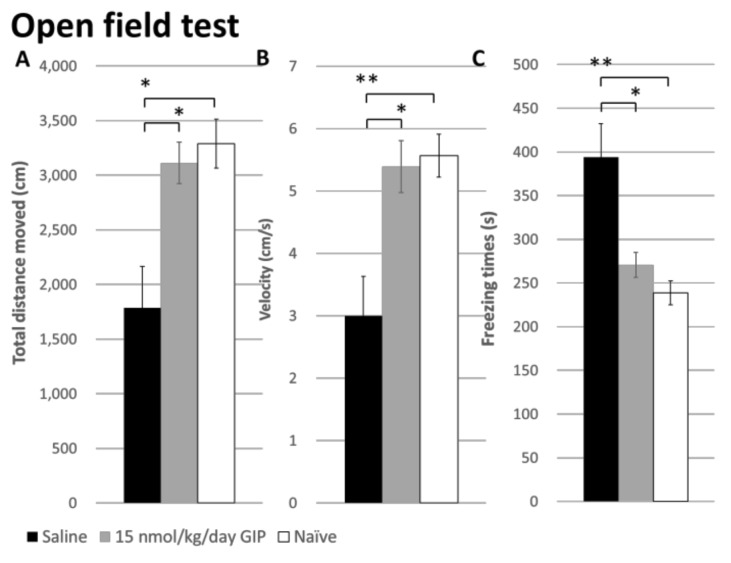

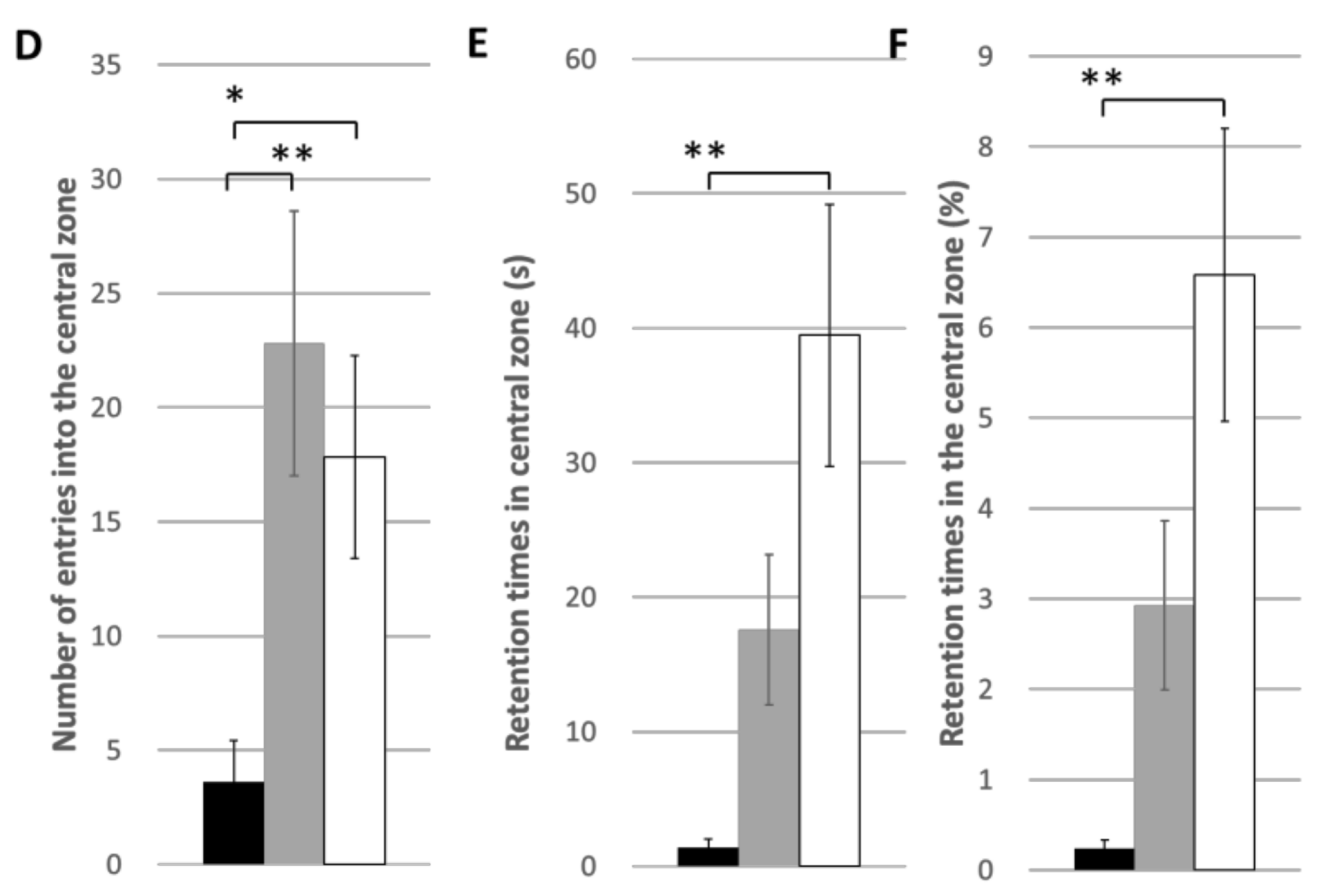
The effects of GIP in the open-field test. (**A**) Total distance moved;(**B**) velocity; and (**C**) freezing times in 10 min; (**D**) The number of entries and (**E**,**F**) retention times into the central zone was significantly higher in GIP treatment group compared to the saline group. (Saline: *n* = 8, GIP15: *n* = 5, Naïve: *n* = 6). All analyses based on two way ANOVA, * *p* < 0.05; ** *p* < 0.01.

Open-field test: We explored the effects of 6-OHDA lesions and GIP treatment on general locomotor activity and anxiety-related behaviors in open field tests (Figure 6). Using one-way ANOVA data analysis, there were significant effects on the total distance moved (*F*(2, 18) = 7.336, *p* = 0.005), velocity (*F*(2, 18) = 7.807, *p* = 0.004), freezing time (*F*(2, 18) = 8.231, *p* = 0.003), number entries into central zone (*F*(2, 18) = 7.322, *p* = 0.008), and retention times in the central zone (*F*(2, 18) = 11.408, *p* = 0.001). A Bonferroni correction for multiple comparisons also revealed significant differences between naïve non-lesioned and 6-OHDA-lesioned animals in the overall parameters: total distance moved, velocity, freezing time, number entries into the central zone, and retention times in the central zone, with adjusted *p*-values of 0.010, 0.009, 0.005, 0.041, and 0.001, respectively. GIP treatment improved locomotor behavior to a level similar to the naïve group.

Furthermore, there was also an effect of GIP treatment on anxiety-like behavior (total distance moved, velocity, freezing time, and number entries into the central zone: *p* = 0.031, 0.021, 0.034, and 0.008) in 6-OHDA rats except for retention times in the central zone (*p* = 0.218). Nevertheless, GIP treatment still trended to improve this deficit in open field tests as well.

## 3. Discussion

In the present study, in a unilateral 6-OHDA MFB lesion rat model of PD, we demonstrated that GIP treatment stabilized apomorphine-induced rotational behavior and showed positive changes in open field locomotor tests, as well as some anxiety-like behaviors. This was achieved at a plasma active GIP concentration that is achievable in humans by use of a DPP-4 inhibitor [24,25]. To our knowledge, our study is the first to evaluate the action of GIP in a 6-OHDA PD animal model and adds to the growing literature that GIP may be of value as a treatment strategy for PD [26,27].

GIP is derived from a 153-amino acid precursor of GIP, pre-proGIP, which is encoded by the GIP gene within the mucosa of the duodenum and jejunum of the gastrointestinal tract. This precursor is processed to GIP by prohormone convertase 1/3, which cleaves out (i) the 42 amino acid-peptide GIP (1–42), that is then stored in secretory granules for release; (ii) a signal peptide; (iii) an N-terminal peptide; and (iv) a C-terminal peptide. All, apart from GIP, appear to be biologically inactive in relation to insulinotropic actions. GIP is distributed into the bloodstream following food ingestion and circulates as a biologically active 42-amino acid peptide (active GIP), but its actions are transient consequent to its rapid hydrolysis by DPP-4 with a resulting half-life of approximately 7 min [17,28]. Of the two endogenous incretins GLP-1 and GIP, GIP is considered to predominantly mediate the incretin effect and post-prandial insulin release in healthy individuals [28,29,30]; however, multiple studies indicate that patients with type 2 diabetes almost completely lose their insulinotropic response to GIP, whereas that to GLP-1 is preserved [28,31]. As a consequence, long-acting, DPP-4 resistant GLP-1R agonists, but not GIP-R agonists, have been developed and clinically approved for the treatment of type 2 diabetes. Whether or not the extra-pancreatic, non-insulinotrophic actions of GIP are retained in other disease states remain to be evaluated [32,33].

Within the brain, GIP is reported to be expressed within multiple neurons and to act as a neurotransmitter [32,33]. Additionally, GIP-R expression has been described across multiple brain regions that include the olfactory bulb, assorted areas within hippocampus, cerebral cortex, substantia nigra, thalamus, cerebellum, and brainstem [34,35], with expression co-localizing with neuronal rather than glial markers. GIP-R activation within the brain is described to induce proliferation of neuronal progenitor cells. This thereby supports neurogenesis [36,37] to provide neurotrophic action, modulate synaptic activity, afford neuroprotective effects against insults such as amyloid-β peptide [20], augment spatial learning and memory [32], and support neuronal regeneration [38]. In relation to PD, preclinically available long-acting GIP analogues have been evaluated in acute and chronic MPTP challenged mice. In these studies, they significantly ameliorated MPTP-induced neuroinflammation, oxidative stress and lipid peroxidation, elevated the expression of BDNF [26,27], reduced markers of dopaminergic loss in striatum and substantia nigra and, in accordance with our 6-OHDA rat study, improved behavioral measures [26,27,33].

The MFB lesion protocol with 6-OHDA used in our study produces a rapid and substantial dopaminergic lesion (>90%), which is greater than that achieved by 6-OHDA striatal dopaminergic lesioning. Hence, the efficacious behavioral effects of GIP might be considered relatively small if compared with other 6-OHDA protocols that do not yield maximal denervation. The progressive increase in apomorphine-induced rotations with repeated weekly tests evident within the saline group in our 6-OHDA MFB study likely is due to increasing sensitization of the denervated dopaminergic receptors. Notably, this sensitization was not seen in the GIP treated group (Figure 5), likely reflecting an augmented dopaminergic input on the lesioned side [27]. In a number of studies involving dopaminergic grafts, an apomorphine challenge showed decreased rotation with repeated tests over time [39]. However, this is seen with lower doses of apomorphine (0.05–0.1 mg/kg) that maximize the difference between supersensitive dopaminergic receptors on the lesioned side compared to the intact side [40]. In any event, the initial levels of apomorphine (0.5 mg/kg)-induced rotations, of over 400 in both lesioned groups in our study, suggest a marked denervation that is characteristic of MFB lesioning with 6-OHDA. The mitigation of 6-OHDA-induced impairments evident in our open field evaluations achieved by GIP further support such neuroprotective dopaminergic actions.

There is well documented cognition and motor dysfunction in PD, with changes in affective, reward, as well as learning or mnemonic behaviors. Further studies with GIP could utilize novel object recognition and Y maze tests to study learning and memory. In addition, studies on reward could utilize conditioned place preference. The Porsolt test can be used to model “depression”. All these tests are readily feasible in mice and can be used for future studies with GIP.

Multiple drug classes have previously demonstrated promising actions in rodent models of PD, but have failed to translate such benefits to human interventional studies [41]. This, in part, has been due to the evaluation of acute drug doses tolerated by rodents that are intolerable in humans [41,42]. Our appraised GIP dose was 15 nmol/kg/day over the course of two consecutive weeks, delivered s.c. under steady-state conditions via an aseptically implanted minipump. This resulted in plasma levels of 203.9 pmol/L total GIP and 12.8 pmol/L active GIP, defined as the high dose within our pharmacokinetic study (Figure 4). An approximately 50% lower dose of 7.8 nmol/kg/day was additionally pharmacokinetically assessed and generated plasma levels of 40.4 pmol/L total GIP and 2.9 pmol active GIP, which is in accordance with total GIP plasma levels (58.6 + 11.8 pmol/L) measured in our prior GIP study [23], in which 38.5 µg/kg/day GIP (equivalent to 7.8 nmol/kg/day) effectively mitigated TBI injury in rats. Notable in our current study, active GIP levels represented only 6% to 7% of total GIP levels across both doses evaluated, and the total and active concentrations achieved proved non-linear in relation to the GIP dose. A 50% GIP dose reduction resulted in an 80% decline in total, as well as active GIP plasma levels. Importantly, these concentrations are achievable in humans following the use of DPP-4 inhibitors [24,25].

GIP has several peripheral actions, with the best known being its glucomodulatory effects. To control for this, blood glucose levels were evaluated herein, together with body weight. Other than postoperative weight loss, which was similar in both lesioned groups, there were no significant effects on body weight. Moreover, there were no changes in blood glucose levels with all animals remaining euglycemic, in accordance with its known glucose-dependent insulinotropic actions and the demonstration that direct infusion of GIP into healthy humans does not affect appetite, energy intake, or energy expenditure [43]. We assume that the GIP effects seen herein to mitigate behavioral deficits associated with dopaminergic lesioning were centrally mediated. Although several previous studies have reported favorable neurotrophic and neuroprotective actions of systemically administered, long-acting preclinical GIP analogues of similar amino acid size to GIP across multiple rodent models of neurodegeneration [6,23,26,27,33,37,44], the proportion of systemic GIP that enters the brain remains unclear. Our recent study of the 39 amino acid GLP-1 incretin mimetic, Exenatide, demonstrated that some 2% of plasma levels reach the brain [16]. This is likely similar for GIP but this warrants measurement in future studies.

**Figure 7:**
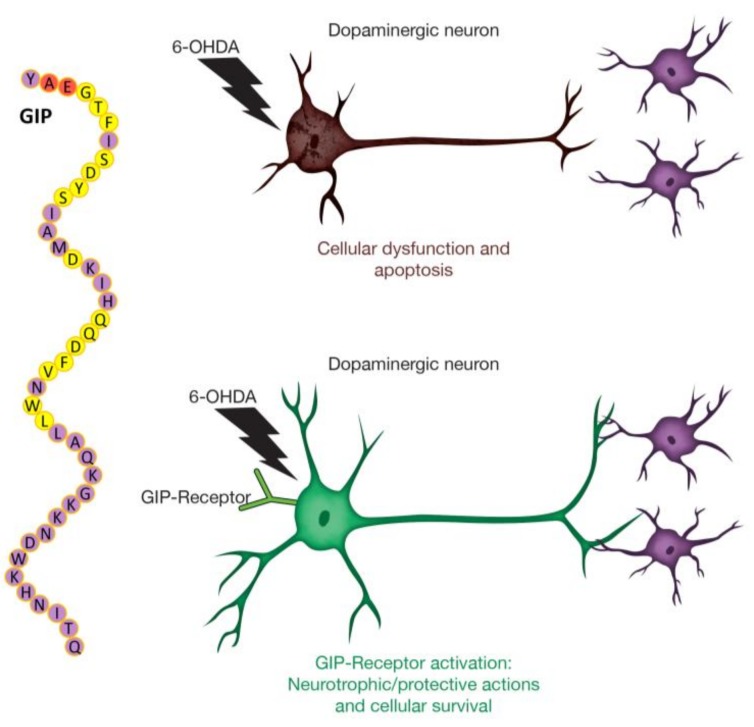
Target engagement and activation of the GIP receptor on dopaminergic neurons provides neuroprotective and neurotrophic actions. It also mitigates 6-OHDA-induced dysfunction, loss of dopaminergic phenotypic features, and cell death. The 42 amino acid sequence of GIP is demonstrated, with the cleavage site for dipeptidyl peptidase-4 (DPP-4) lying between the 2nd and 3rd N-terminal amino acids highlighted in red. The DPP-4 mediated cleavage of GIP results in a loss of pharmacological activity. Amino acids shown in yellow are homologous with the structure of glucagon.

GIP, similar to GLP-1, induces cAMP production via binding and activation of its cognate receptor on neurons [45]. As cAMP is the first intermediate in the neurotrophic CREB pathway, it may be inferred that the neurotrophic and neuroprotective effects of both incretins are similarly mediated via the activation of the protein kinase A (PKA) and PI3K/AKT signaling pathways [7,37]. These pathways regulate numerous downstream targets, including glycogen synthase kinase 3 β (GSK3-B) and FOXO1, which are involved in pathological processes underlying PD, thereby promoting cell survival [15,46]. Albeit, as noted above, there are no clinically available long-acting GIP analogues; however, there are reports of the co-expression of GLP-1R and GIPR on the same cells [38,47], supporting the potential for synergistic actions as either homomers or heteromers [48] and the use of dual (Twincretin) incretin agonists [49]. Such single peptide dual agonists have recently demonstrated promising actions in animal models of neurodegeneration [45,50], including PD [51,52]. Our current studies add to the increasing evidence that the augmentation of GIP signaling may be of clinical value for PD and other neurological disorders, warranting further investigation. A schematic diagram of GIP mechanisms is shown in Figure 7.

## 4. Materials and Methods

### 4.1. Animal Handling and Preparation

Adult male Sprague-Dawley (SD) rats (270 to 340 g) were obtained from LASCO (Taipei, Taiwan) or from Taconic (Germantown, NY, USA) for use in studies performed at Taipei Medical University and the Intramural Research Program of the National Institute on Aging. Rats were provided food and water ad libitum and were maintained on a 12 h light/dark cycle in temperature- and humidity-controlled animal centers at both institutions. All experiments complied with the standard of the Institutional Animal Care and Use Committee, Taiwan (LAC-2016-0202) or (ii) the Animal Care and Use Committee of the Intramural Research Program, National Institute on Aging (438-TGB-2016). These studies were also in compliance with the guidelines for animal experimentation of the National Research Council (Committee for the Update of the Guide for the Care and Use of Laboratory Animals, 2011) and the National Institutes of Health (DHEW publication 85–23, revised, 1995). A minimal number of rats were used for each study and all efforts were made to minimize any potential suffering. Protocol number, LAC-2016-0202, approved on 19 September 2016.

### 4.2. Treatment Groups

Modified surgical procedures were based on our previously described methods [53]. Under Zoletil-Rumpun (10 mg/kg: 2 mg/kg, *i.p.*) anesthesia, SD rats were placed in a stereotaxic frame with the nose bar positioned 3.3 mm below the interaural line. Unilateral 6-hydroxydopamine (6-OHDA, 8 μg dissolved in 0.9% saline with 0.02% ascorbic acid) was injected for DA lesions into the left median forebrain bundle (MFB, coordinates: anteroposterior [49,53], −4.3 mm; lateral (L), 1.6 mm from bregma; ventral (V), 8.2 mm from the dura).

Human GIP or a saline vehicle was delivered by a *s.c.* ALZET micro-osmotic pump that was implanted aseptically under anesthesia 2 days pre-lesion (ALZET 2002, Durect Corp., Cupertino, CA, USA). This delivered GIP over the course of 2 weeks at a rate of 15 nmol/kg/day. In this study, all PD rats were divided into 2 groups treated with either saline or GIP.

### 4.3. Body Weight

All rats were time-dependently weighed throughout the study.

### 4.4. Fasting Blood Glucose

After 12 h fasting, blood glucose from the rats in both groups was measured using an ACCU-Check glucose meter (Roche Diagnostics, Indianapolis, IN, USA).

### 4.5. GIP Plasma Assay

Plasma concentrations of GIP were quantified in a parallel series of SD rats similarly implanted with a *s.c.* ALZET micro-osmotic pumps containing either a saline vehicle or 7.8 or 15 nmol/kg/day GIP. Animals were euthanized at 5 days after pump insertion. Blood was taken by cardiac puncture and immediately placed into iced heparinized tubes containing an excess of DPP4 inhibitor. These were directly centrifuged (1500× *g* at 4 °C). The plasma was then removed and immediately frozen to −80 °C. Samples were later thawed on wet ice and analyzed for human active GIP (1–42) (Immuno-Biological Laboratories, Gunma, Japan) by ELISA and for total GIP concentrations (Meso Scale Discovery, Rockville, MD, USA)

### 4.6. Apomorphine-Induced Rotation

Apomorphine (0.5 mg/kg in 0.1% ascorbic acid, s.c.)-induced contralateral rotations were recorded using a digital video camera to count the number of rotations for 60 min at weekly intervals up to 4 weeks post-lesion (i.e., 7, 14, 21, and 28 days post-lesion) using the same test conditions. The net number of rotations was calculated (contralateral minus ipsilateral full turns). In unilaterally 6-OHDA lesioned animals, apomorphine-induced contralateral rotation is a well-established correlate of dopamine depletion, with rotation levels seen here indicative of an 80–90% loss [53].

### 4.7. Open-Field Tests

In addition to its well-known motor manifestations, PD is also known to elicit neuropsychiatric disorders such as anxiety [54,55]. We thus used open field measurements in the lesioned animals, as well as in intact animals, to evaluate not only general motor activity but also anxiety-related exploratory activity [56,57]. Rats were first habituated to an open field Plexiglas arena (60 × 60 × 100 cm) for 10 min at day 4 and 5 post-lesion. At day 6 post-lesion, locomotor activity and anxiety-like behavior were monitored for 10 min using open field tests. The rat behaviors were recorded using an EthoVision system. Total distance, velocity, and freezing times in the open-field were considered general locomotor activities. The number and retention times in the central zone of the open field were measured as anxiety-like behaviors. The center of the open field was defined as a 30 × 30 cm^2^ in the geometric center of the arena.

### 4.8. Overview of Experimental Design and Statistical Analyses

In summary, we investigated the effects of human GIP on overall body weight, blood glucose, as well as behavioral changes in open-field tests and drug-induced rotational behavior, after inflicting 6-OHDA lesions of the nigrostriatal pathway, at multiple time points post-lesion (Figure 1). Animals were divided into saline, and 15 nmol/kg/day GIP groups. Two days prior to the 6-OHDA lesion, rats were treated with saline or 15 nmol/kg/day GIP over the course of two consecutive weeks delivered subcutaneously under steady-state conditions via aseptically implanted mini pumps, as detailed above.

Data was analyzed using SPSS (version 11.0) and are expressed as mean + standard error of the mean values The effects of GIP on PD were evaluated by a one-way repeated measures analysis of variance (ANOVARM), with “group” as a between-subjects factor and “time” as a within-subject factor. For the long-term effects between-subjects factor, an independent *t*-test was analyzed on the follow-up data. The Bonferroni post hoc test was used to compare between groups, as required. Effects were considered to be significant if *p* < 0.05.

## 5. Conclusions

GIP pretreatment, at a clinically translatable dose, reduced the progressive DA receptor supersensitivity induced by unilateral injection of 6-OHDA into the MFB. In addition, locomotor and anxiety-like behavioral changes were also ameliorated by GIP. Thus, GIP receptor agonists, together with GLP-1 receptor agonists, may be a future therapeutic target for PD.

## Abbreviations

PD	Parkinson’s Disease
6-OHDA	6-Hydroxydopamine
TH	Tyrosine Hydroxylase
DA	Dopaminergic
SN	Substantia Nigra
MFB	Medial Forebrain Bundle
